# Editorial: Understanding the Link Between the Developing Brain and Behavior in Adolescents

**DOI:** 10.3389/fnhum.2021.663454

**Published:** 2021-05-07

**Authors:** Tim J. Silk, Megan M. Herting, Lara M. Wierenga, Nandita Vijayakumar

**Affiliations:** ^1^School of Psychology, Faculty of Health, Deakin University, Melbourne, VIC, Australia; ^2^Department of Preventive Medicine, Keck School of Medicine, University of Southern California, Los Angeles, CA, United States; ^3^Institute of Psychology, Leiden University, Leiden, Netherlands; ^4^Leiden Institute for Brain and Cognition, Leiden, Netherlands

**Keywords:** adolescence, childhood, brain development, brain behavior correlation, neurodevelopment, neuroimaging

The transition through adolescences marks one of the most dynamic and influential periods of brain development. This period of neural maturation and reorganization drives a myriad of cognitive, social, and behavioral changes, which ultimately lay the foundations for successful adult functioning. However, this period of neurodevelopment also opens up increased vulnerability to affective and behavioral dysregulation, with a dramatic rise in the incidence of mental illness during adolescence. It is thus important to examine when, where, and why maturational changes in brain structure and function occur, in order to better understand cognition and behavior in both typical and atypical populations. In this Research Topic, we bring together a collection of the latest in neuroimaging research and reviews to understand the link between the developing brain and behavior in adolescents.

We start with two articles that examine the influence of the environment we grow up in on structural brain development. Gonzalez et al. showed that socioeconomic status (SES), measured using the income-to-needs ratio (INR), was positively related to cortical surface area in 9-10-year-olds in the Adolescent Brain and Cognitive Development cohort—a large (*N* = 8158) diverse sample of the US population. These associations were non-linear such that incremental increases in INR had the largest effect on both surface area and cognitive function in those living in deep poverty. Moreover, access to economic and social resources contributed to better cognitive performance in these children, highlighting the importance of public health policies to invest in these resources and support healthier outcomes. McLachlan et al. also showed that SES (based on parental education and occupation) was positively related to limbic volumes in their sample of neurotypical controls aged 7-19 years old (*N* = 70), but failed to identify such associations in an age-matched group with perinatal alcohol exposure (PAE, *N* = 69). It is argued that these children and adolescents may have suffered early PAE-related injury that overwhelmed postpartum brain development, such that it reduced neural plasticity or sensitivity to SES. Thus, family SES may have varied developmental effects across different populations.

Much of what is known about the adolescent brain is based on cortical gray matter, as assessed by structural morphology or task-based function. However, the connecting white matter continues to undergo a number of microscopic neurobiological processes. Beaulieu et al. advance a broad literature on the role of inefficient neuronal communication on reading ability using advanced imaging techniques that inform us about specific tissue microstructural properties of white matter. They use myelin water fraction imaging in a small sample (*N* = 20) of 10-18-year-olds, and provide preliminary evidence that lower myelin content differentiates poor from good readers. Findings suggest that poor myelination, and thus decreased conduction speed along axons, amongst key reading circuitry contributes to reading ability; with these findings having broader significance for cognitive and academic development in children and adolescents.

In order to understand common behaviors seen in adolescence, two studies took approaches to examine hypotheses regarding the disconnect between prefrontal control systems and the limbic reward system. Kim et al. collected measures of decision-making performance on a computer-based food choice task and obesity in a sample of 71 8-22-year-olds. Specially targeting prefrontal cortical thickness and volume of amygdala nuclei, they found metrics of obesity were associated with gray-matter structure in prefrontal cognitive control regions and the central nucleus of the amygdala as part of the reward system. Furthermore, these structures were predictive of measures of dietary self-control. In a small sample (*n* = 19), Tymofiyeva et al. examined white-matter connectivity of the nucleus accumbens, anterior cingulate, and amygdala to elucidate potential neural correlates of smartphone dependence in adolescents. Higher structural connectivity of the amygdala with other brain regions (node centrality) was positively correlated with smartphone dependence, which the authors suggest may reflect the amygdala becoming over-sensitized with repetitive smartphone use and its associated rewards. They suggest a potential mediating link between excessive smartphone use and mental health problems through sleep problems. However, both these cross-sectional studies stress that causal direction cannot be determined and call for longitudinal studies.

A couple of comprehensive reviews summarize the potential for neural changes in adolescents both in a negative capacity, such as a consequence of substance use, and in a positive capacity with training-induced plasticity. Hamidullah et al. review findings on neural, behavioral, and cognitive changes associated with substance use, focusing on the most commonly used substances (nicotine, alcohol, cannabis, and opioids) as well as their combined use. In addition to the increased risk of future substance use, drug use in adolescence can negatively impact ongoing brain development, contributing to a heightened risk of cognitive deficits and psychopathology. Tymofiyeva and Gaschler present a systematic review of neuroimaging research on training-induced changes in neural structure and function. They identified a diverse set of empirical studies examining varied developmental populations (including ADHD, autism, dyslexia, and dyscalculia) and utilizing different training interventions, study designs, and MRI modalities, showing support for the generalized effect of training-induced changes in neural plasticity in young people. The authors discuss important limitations in the literature, including the prevalence of under-powered studies, and outline recommendations for future research to advance the translational applicability of this field.

Finally, in light of the prevalence of mental health issues that arise during adolescence, in their perspective article, Tymofiyeva et al. summarize recent structural and functional connectivity research that has shed light on the development of psychiatric disorders in adolescence, and present a vision and roadmap for the future, stressing the need for brain-based diagnosis and personalized care. As part of this roadmap the authors make recommendations, with a cost-benefit analysis, of a brief routine neuropsychological and neuropsychiatric imaging (NPPI) protocol for every adolescent, that can be compared to normative data to directly inform clinicians and assist with the early detection of psychiatric disorders.

In summary, this collection of articles demonstrates how emerging and advancing neuroimaging techniques are enabling the greater understanding of the link between the developing brain and the behaviors seen in adolescents, and expresses exciting potential for the future in this burgeoning field.

## Author Contributions

TS and NV wrote the first draft. All authors revised and edited the manuscript.

## Conflict of Interest

The authors declare that the research was conducted in the absence of any commercial or financial relationships that could be construed as a potential conflict of interest.

